# Treatment of ventriculoperitoneal shunt infection and ventriculitis caused by *Acinetobacter baumannii*: a case report

**DOI:** 10.1186/s13256-018-1680-5

**Published:** 2018-05-23

**Authors:** Gebre Teklemariam Demoz, Minyahil Alebachew, Yirga Legesse, Belete Ayalneh

**Affiliations:** 1 Clinical Pharmacy and Pharmacy Practice Unit, Department of Pharmacy, College of Health Sciences, Aksum University, Aksum, Ethiopia; 2 Department of Pharmacology and Clinical Pharmacy, School of Pharmacy, College of Health Sciences, Addis Ababa University, Addis Ababa, Ethiopia; 3 Department of Clinical Pharmacy, School of Pharmacy, College of Health Sciences, Mekelle University, Mekelle, Ethiopia

**Keywords:** *Acinetobacter baumannii*, Ventriculitis, Hydrocephalus, Ventriculoperitoneal shunt, Extra ventricular drainage (EVD), Multidrug resistance (MDR), Pandrug-resistant (PDR)

## Abstract

**Background:**

*Acinetobacter baumannii (A. baumannii*) infections are a recognized problem in healthcare, causing ventriculoperitoneal shunt infection and ventriculitis. Such infections are serious intracranial infection that can lead to serious complication and death. Treatment of infection caused by *A. baumannii* becomes difficult because of its inclination to develop pandrug resistance to the universally used antibiotics. In this case, we focused on pediatric ventriculitis/shunt infection caused by *A. baumannii* in an extensive follow-up and report the subsequent treatment outcome. Very limited information regarding the therapeutic options against *A. baumannii* ventriculitis/shunt infection is available in our hospital. Thus, we present one such case and the problems in its treatment.

**Case presentation:**

We reported the case of a 6-year-old Ethiopian boy who developed ventriculitis/shunt infection from the pandrug-resistant strain of *A. baumannii*, after decompression of a craniotomy for medulloblastoma. Following the surgical procedure, he had developed hydrocephalus and ventriculoperitoneal shunt infection/ventriculitis as he presented with persistent fever, elevated white blood cell count, reduced glucose level, and the cerebrospinal fluid culture revealed *A. baumannii*, which was not responding to most of commercially available antibiotics systemically. Our patient was successfully treated with intravenous ampicillin-sulbactam.

**Conclusions:**

We presented our case of pandrug-resistant *A. baumannii* ventriculoperitoneal shunt infection and ventriculitis successfully treated with a systemic ampicillin-sulbactam. Provision of systemic ampicillin-sulbactam should not be undermined. Therefore, this case exemplifies that intravenous administration of ampicillin-sulbactam can be a good therapeutic option against *A. baumannii* ventriculoperitoneal shunt infection and ventriculitis.

**Electronic supplementary material:**

The online version of this article (https://doi.org/10.1186/s13256-018-1680-5) contains supplementary material, which is available to authorized users.

## Background

Cerebrospinal fluid (CSF) shunt devices are commonly indicated for the treatment of hydrocephalus in children, and have served to improve survival and reduce mortality. However, ventriculoperitoneal shunt infection is a serious complication, commonly with an underlying bacterial etiology [1, 2]. *Acinetobacter baumannii (A. baumannii*) infections are a recognized problem in healthcare, causing ventriculoperitoneal shunt infection and ventriculitis. The incidence of *A. baumannii* resistance is increasing across the globe, and that makes it difficult to treat in neurosurgical practice. It can cause neurological defects, and can lead to a long course of treatment and hospitalization. Such infections are serious intracranial infection that can lead to death [3, 4].

Treatment of ventriculoperitoneal shunt infected with *A. baumannii* is curious because of its inclination to develop pandrug resistance (PDR) to commonly used potent antibiotics in healthcare situations. PDR is defined as when an isolate exhibits resistance to all seven antipseudomonal antimicrobial agents; included antipseudomonal penicillins, cephalosporins, carbapenems, monobactams, quinolones, aminoglycosides, and polymyxins [5]. The Infectious Diseases Society of America (IDSA) recommends vancomycin plus an antipseudomonal β-lactam (such as cefepime, ceftazidime, or meropenem) as empiric therapy for healthcare-associated ventriculitis. For treatment of infection caused by *A. baumannii*, meropenem is strongly recommended; for strains that demonstrate carbapenem resistance, intravenous and/or intraventricular colistin is recommended [6]. In *A. baumannii* ventriculitis, the major problems provoking clinicians in intensive care units are related to the severity of the bacteria and its extraordinary ability to develop multiple resistance mechanisms against major classes of available antibiotics [7].

Little is known regarding the appropriate treatment of ventriculoperitoneal shunt infection and ventriculitis in pediatric patients. Hence, early diagnosis and effective treatment is compulsory. Our primary purpose was to report the successful treatment of ventriculoperitoneal shunt infection and ventriculitis caused by *A. baumannii* with ampicillin-sulbactam. Therefore, this case may be helpful in reminding clinicians to consider ampicillin-sulbactam treatment in the management of cases infected with *A. baumannii*. Thus, this case could also contribute important information for physicians facing such complicated infections.

## Case presentation

A 6-year-old Ethiopian boy was admitted to the pediatric intensive care unit of the Black Lion Specialized Hospital with medulloblastoma and hydrocephalus. He was the first child in his family and came from low socioeconomic status. No history of birth defect, head injury, trauma, central nervous system infections, malignancy and related conditions, except that a year prior, he had been admitted in the private hospital intensive care unit, for more than a month; a ventriculoperitoneal shunt was placed for treating hydrocephalus but there was no recorded history of infections and complications. He presented in our hospital with high-grade global type of headache, poor appetite, nausea, vomiting, tiredness, decreased vision, and drowsiness. These are typical signs and symptoms for medulloblastoma. However, at admission he had no fever, cough, or chest pain. He was also conscious, with a Glasgow Coma Scale (GCS) of 15/15, with no motor deficit or sensory disorder. On admission, his blood pressure (BP) was 140/85 mmHg, with a regular heartbeat of 60 beats per minute, heart sounds were clearly auscultated with no murmur, there was no murmur of mitral insufficiency or ventricular defect, no signs of heart failure, and he was without edema of his lower limbs. His peripheral pulses were perceived symmetrically. The rest of the physical examination was also normal.

**Fig. 1:**
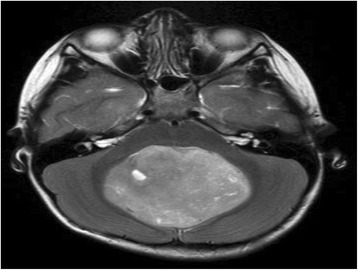
Contrast magnetic resonance imaging revealing an enhancing posterior fossa mass, involving the third ventricle: medulloblastoma

On admission, a brain computed tomography (CT) scan and brain magnetic resonance image (MRI) was performed and indicated the presence of medulloblastoma (a mass that was involved the posterior fossa, Fig. 1). An immediate craniotomy procedure was performed for the removal of the mass. A biopsy was also performed and revealed no malignancy or spread of brain tumor. The radiologist and neurologist team suggested that no further treatment was needed, radiotherapy or chemotherapy. However, following the craniotomy procedure, the neurological findings progressively deteriorated. This showed he had developed obstructive hydrocephalus as he presented with gait disturbance, low attention, seizure, and uncontrolled body movement with an elevated intracranial pressure (ICP). To restore this CSF circulation within both lateral ventricles, a ventriculoperitoneal (VP) shunt was placed. However, his mental status could not be improved. Thus, on the 4th day, the VP shunt was replaced with external ventricular drainage (EVD) which was undertaken to control the elevated ICP because of hydrocephalus.

On the 6th of the surgery, he became comatose with a GCS score of 7/15 and hemiparesis. He needed nasogastric tube (NGT) feeding for nutrition. Meanwhile, he presented with high-grade fever (39.8 °C), severe headache, vomiting, lethargy, and deteriorating consciousness. The CSF examination revealed an elevated white blood cell (WBC) count of 21.0 (10^9^) cells/L), reduced blood sugar (29 mg/dL), and elevated protein level (219 mg/dL).

A blood culture specimen was drawn from his CSF. Awaiting the culture results, an empiric antibiotic therapy was initiated with an intravenous dose of vancomycin and piperacillin-tazobactam. However, he did not respond to this regimen. The second (a day after) CSF result showed that a glucose concentration of 32 mg/dL, a protein level of 260 mg/dL, and a WBC cell count of 20.0 (10^9^) cells/L). The gram staining of the CSF had also shown no organisms. Seven days after surgery, blood culture revealed no growth.

However, the clinical condition of the patient still did not improve. The vancomycin and piperacillin-tazobactam regimen was changed to cefepime and gentamycin for 7 days. Later, those regimens were also switched to intravenous ciprofloxacin and metronidazole. Despite taking these medications; his fever still persisted and a poor clinical response was observed. Thus, the CSF analysis was repeated on the 18th day of illness and revealed a glucose concentration of 21 mg/dL, a protein level of 200 mg/dL, and a total WBC cell count of 24.0 (10^9^) cells/L). The CSF smear for Gram statin did not reveal any bacteria. However, the patient still had fever; low consciousness level, a low glucose level, and an elevated protein level were noted.

**Fig. 2:**
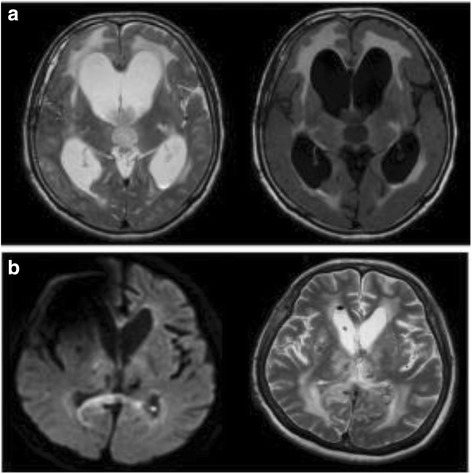
**a** Magnetic resonance imaging on the 25th day of admission shows abnormal hyperintensities in the trigones of both lateral ventricles, suggesting debris (*left panel*). The central panel and fluid-attenuated inversion recovery imaging (*right panel*) show abnormal periventricular intensities. **b** During discharge magnetic resonance imaging. Both ventriculitis and hydrocephalus have been resolved

On the 25th day of admission, along with the ventriculoperitoneal shunt infection the MRI showed evidence for ventriculitis, including intraventricular debris in the ventricles on diffusion-weighted imaging and abnormal periventricular intensities on fluid-attenuated inversion recovery imaging (Fig. 2a). Examination of his CSF revealed a persistent elevation of his white blood cell count and reduction in glucose level. This was confirmed with one CSF culture along with a positive Gram stain, which was generated by *A. baumannii.* Although the shunt system was removed immediately and antibiotic treatment was initiated, the infection persisted despite intravenous administration of antibiotics including vancomycin and piperacillin-tazobactam, cefepime with gentamycin, followed by ciprofloxacin with metronidazole. The bacteria was resistant to all the antibiotics examined in the laboratory by disk diffusion susceptibility test, including β-lactamase inhibitor penicillins, carbapenems, cephalosporins, fluoroquinolones, and aminoglycosides. However, sensitivity to colistin, aztreonam and tigecycline was not tested because of it was unavailable in our hospital.

Later, with the organism identified; the intravenous antibiotic therapy was continued with ampicillin-sulbactam 1.3 g every 6 h. For CSF isolates of *A. baumannii,* the minimum inhibitory concentration (MIC) of ampicillin-sulbactam was 4 μg/mL (details of each antibiotic MIC is stated in Additional file 1: Tables S1 and S2). Our patient’s fever begun to subside and he became afebrile on the 3rd day of the systemic therapy with ampicillin-sulbactam. The CSF culture was performed and was negative on the 10th day of this systemic therapy, and the CSF cell count had reduced to 15.0 (10^9^) cells/L). On the 14th day of the therapy with ampicillin-sulbactam, the CSF culture was completely sterile, the CSF cell count had also dropped to 9.0 (10^9^) cells/L), and the therapy was discontinued. The gradual improvement and clinical outcome of our patient was noted. Our patient’s level of consciousness rapidly improved. The overall condition of the child was improved oddly and progressively, he began to feed orally and became more collaborative. Three consecutive blood culture analyses were performed and revealed no microbial growth all over. Indicating that the infection had gradually subsided and had been cured.

Furthermore, no complications or side effects were witnessed during the treatment. The clinical status of our patient was progressively and remarkably improved. Meantime, after a month the surgical team had diverted the EVD to the left side. Finally, our patient was discharged from the hospital in a stable and good condition. Our patient has been followed up for 2 months after being discharged from the pediatric ward as ambulatory. We found no evidence of recurrence of infection or any neurological defects or malfunction had occurred as of 6 months postoperatively (Fig. 2b). The patient is now able to walk without mobility aids, eat meals by himself, and live independently at home.

## Discussion

We reported the clinical feature and treatment outcome of a single case of a patient with *A. baumannii* infection. Our case illustrated that the separation of a pandrug-resistant mostly for the Gram-negative clinical specimens does not necessarily mean a depraved outcome. This may be clarified by numerous reasons. First, the achieved concentration of several antimicrobial agents in some body fluids, including urine may exceed the minimal inhibitory concentration of the isolated pathogen. Second, infections, even severe ones, are sometimes are self-limiting without the use of antimicrobial agents, as the preantibiotic era taught us [8]. Third, pandrug-resistant bacteria may be immigrants in patients receiving several classes of antimicrobial agents for a long period of time, while the real pathogen may not be isolated. Fourth, it has been shown that occasionally multi drug resistance (MDR) organisms may exhibit decreased virulence compared to other more sensitive organisms of the same species [9].

In the management of resistant Gram-negative bacterial infections, combination of colistin with β-lactam antibiotics, may be a useful agent [5]. Even though, there are already clinical isolates of Gram-negative bacteria that are resistant to all available antibiotics, infections due to resistant Gram-negative bacteria continue to increase. This led to looking at other therapeutic options for these infections, such as colistin and ampicillin-sulbactam combination [10]. However, colistin is not commercially available in our hospital and this led us to use such many of the antibiotics systemically. Finally, we were compelled to use beta-lactam alone. But the treatment outcome of our case was found to be successful. Thus, our case was found to be in line with cases reported of six patients, who were infected with *A. baumannii* meningitis, which were effectively cured with ampicillin-sulbactam [11].

One study also reported that the alternative choice drug for the treatment of *A. baumannii* infections resistant to carbapenems strains were parenteral co-administration of β-lactam ampicillin with the β-lactamase inhibitor sulbactam, (ampicillin-sulbactam) [12]. Of the β-lactamase inhibitors, sulbactam possesses the greatest intrinsic bactericidal activity against *A. baumannii* isolates [13]. The prevalence of drug-resistant strains is increasing, and treatment options are increasingly limited. Effective therapy remains likely when the organism is proven to be susceptible. Thus, a single testing method may result in incorrect susceptibility results and lead the clinician to select a potentially ineffective agent. The challenge for the clinician is to combine local susceptibility patterns with the agents that are most likely to be effective. The use of combination therapy, whether empirical or targeted, has yet to be demonstrated [10]. Despite the partiality for treatment of ventriculoperitoneal shunt infections with antibiotics alone, this mode of treatment was usually not successful; survivors had continued or recurrent infection. Thus, patients with ventriculoperitoneal shunts were treated more frequently by early removal of the shunt followed by administration of systemic and/or local antibiotics [1].

*A. baumannii* remains an important and difficult-to-treat pathogen whose resistance patterns result in significant challenges for the clinician. Despite the prevalence and interest in *A. baumannii* infections, there is relatively limited well-controlled scientific data to help the clinician in selecting the optimal empirical and subsequent targeted therapy for a variety of infections. Indeed, the pharmacotherapeutic effect of such microorganisms may also need a special attention in neurosurgical cases, while the clinician’s proficiency is required to review them based on local and clinical practice.

Numerous studies reported that *A. baumannii* were multidrug-resistant, which makes it challenging to cure using commercially available potent antibiotics. However, we treated our patient successfully with ampicillin-sulbactam intravenously. There were neither recurrences nor superinfections. Thus, eradication of *A. baumannii* from the CSF in shunt-associated ventriculitis required a complete removal of ventricular devices followed by administration of bactericidal antibiotics.

According to IDSA recommendation, patients with ventriculitis should be monitored for response to treatment based on clinical parameters. For those patients with ventriculitis and an external drainage device, monitoring of CSF cultures is also recommended to ensure that they become negative [14]. In our case, multiple monitoring parameters were performed in order to confirm our patient response to the current treatment modalities. In addition, additional CSF analysis was performed to ensure that the CSF parameters had improved and the cultures revealed negative results.

Therefore, we recommend that intravenous combination of ampicillin-sulbactam can be used in such severe and life-threatening infections caused by *A. baumannii* resistance in which there are no available other therapeutic options. This indicates that ampicillin-sulbactam is a good and safe therapeutic option to treat ventriculoperitoneal shunt infection and ventriculitis caused by multidrug-resistant *A. baumannii*. We observed that administration of systemic ampicillin-sulbactam therapy for hydrocephalus shunt infection and ventriculitis caused by *A. baumannii* may be effective in reducing the rate of further neurological complication and/or defects and the duration of hospitalization. Moreover, we hope future case studies will explicate a number of definitive and practical options.

## Conclusion

We reported the outcome of a single case of a patient with *A. baumannii* infection treated with ampicillin-sulbactam and our difficulties in patient management. This drug could be a treatment option against *A. baumannii* when colistin is not available. In our practice, we successfully treated a patient with pandrug-resistant *A. baumannii* ventriculoperitoneal shunt infection and ventriculitis with a systemic ampicillin-sulbactam. As a conclusion, an intravenous administration of ampicillin-sulbactam could be an effective therapeutic option in the treatment of ventriculoperitoneal shunt infection and ventriculitis caused by *A. baumannii* resistance to most potent antimicrobials, including carbapenem and other β-lactam drugs. Therefore, even though *A. baumannii* is resistant to most other potent antimicrobials, systemic provision of ampicillin-sulbactam should not be undermined.

## Additional file

Additional file 1: **Table S1.** Medical conditions and pattern of interventions and prescribed antibiotics. **Table S2.** Sensitivity tests performed and results. (DOCX 21 kb)
