# Supplementary material for: Treatment of ventriculoperitoneal shunt infection and ventriculitis caused by Acinetobacter baumannii: a case report
Source: J Med Case Rep. 2018 May 23;12:141. doi: 10.1186/s13256-018-1680-5 (PMC5964891; doi:10.1186/s13256-018-1680-5)
Supplement: Supplementary file 1 — Table S1. Medical conditions and pattern of interventions and prescribed antibiotics. Table S2. Sensitivity tests performed and results. (DOCX 21 kb) [file 13256_2018_1680_MOESM1_ESM.docx]

**Table S1:** **Medical conditions and pattern of interventions and prescribed antibiotics**

| Medical conditions | Drug or intervention | Dosage regimen  (dose, route, frequency) |  | Outcome(response) |
| --- | --- | --- | --- | --- |
| Medulloblastoma | Craniotomy | Surgery(removal) |  | Good |
| Hydrocephalus | VP-shunt placed | Draining CSF Fluid |  | Infection |
| Hydrocephalus | EVD | Draining CSF Fluid |  | Good response |
| Ventriculitis and  VP shunt infection | Ceftriaxone(prophylaxis) | 450mg IV every 12 hours |  | Poor |
|  | Vancomycin plus | 300mg IV every 8 hours |  | Poor |
|  | Piperacillin-Tazobactam | 1.5gram IV every 8 hours |  | Poor |
|  | Metronidazole plus | 170mg IV every 8 hours |  | Poor |
|  | Ciprofloxacin | 150mg IV every 8 hours |  | Poor |
|  | Gentamycin plus | 50mgIV every 8 hours |  | Poor |
|  | Cefepime | 800mg IV every 8 hours |  | Poor |
|  | Ampicillin-sulbactam | 1.3 gm IV every 6 hours |  | Cured |

VP: ventriculoperitoneal, EVD: Extra ventricular drainage, CSF: Cerebrospinal fluid, IV: Intravenous, mg: milligram

**Table S2: Sensitivity tests performed and results**

| Medication | Susceptibility | MIC | Medication | Susceptibility | MIC |
| --- | --- | --- | --- | --- | --- |
| Meropenem | R | ≥8µg/ml | Vancomycin | R | ≥16µg/ml |
| Piperacillin-tazobactam | R | ≥4µg/ml | Gentamycin | R | ≥16µg/ml |
| Ceftriaxone | R | ≥64µg/ml | Amikacin | R | ≥64µg/ml |
| Ceftazidime | R | ≥32µg/ml | Tobramycin | R | ≥16µg/ml |
| Cefepime | R | ≥32µg/ml | Tigecycline | Not done | N/A |
| Ampicillin-sulbactam | S | ≤4µg/ml | Colistin | Not done | N/A |
| Ciprofloxacin | R | ≥4µg/ml | Aztreonam | Not done | N/A |

MIC: minimal inhibitory concentration, R: resistant, S: susceptible, N/A: Not available, µg: microgram, ml: milliliter
